# Application of artificial intelligence in osteoporosis: a review

**DOI:** 10.3389/fmed.2025.1718554

**Published:** 2025-12-12

**Authors:** Shu-Ting Fan, Min Lu, Jin-Lu Dong, Yi-Lin Li, Li-Na Hao, Ren-Chao Dong, Ming-Dong Hou

**Affiliations:** 1 School of Information Science and Electrical Engineering (School of Artificial Intelligence), Shandong Jiaotong University, Jinan, China; 2 Department of Pharmacy, The Fourth People's Hospital of Jinan, Jinan, China; 3 Department of Traditional Chinese Medicine, Yantai Zhifu District Maternal and Child Health Hospital, Yantai, China; 4 Department of Pharmacy, Children’s Hospital Affiliated to Shandong University (Jinan Children’s Hospital), Jinan, China

**Keywords:** artificial intelligence, deep learning, interdisciplinarity, osteoporosis, bone metabolism, precision medicine

## Abstract

Osteoporosis is a systemic skeletal disease defined by decreased bone mass, deteriorated bone microarchitecture, and increased fracture susceptibility, all of which substantially increase the risks of functional disability and overall mortality. Given its complex pathophysiology, chronic progression, and frequently asymptomatic clinical presentation, the diagnosis and treatment of osteoporosis present multiple challenges. Artificial intelligence (AI) technologies integrate radiomics and multi-omics data to develop intelligent frameworks that map disease progression, identify molecular biomarkers, and stratify individual risk, offering new strategies for molecular-level support in precision diagnosis, preventive monitoring, and personalized intervention. Notably, AI-driven models for drug target prediction and therapeutic response evaluation have also been developed, offering mechanism-based strategies for novel drug discovery. This study outlines how AI advances osteoporosis management by applying technological breakthroughs in foundational and clinical research across key areas, including optimizing preventive monitoring, developing precision diagnostics, supporting personalized treatment decisions, and enabling molecular-targeted drug discovery, thereby offering a strong theoretical basis and practical pathways within precision and personalized medicine.

## Graphical abstract

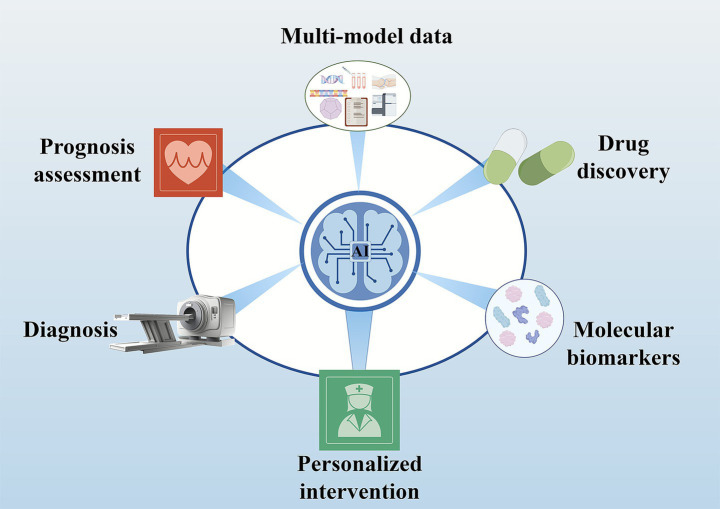

## 1 Introduction

Osteoporosis (OP) is a systemic metabolic bone disease marked by low bone mineral density (BMD), deteriorated bone micro-structure, and increased fragility, which significantly raises the risk of fractures (1–4). With the population, osteoporosis has become a major public health issue. According to the International Osteoporosis Foundation (IOF), about 18.3% of people currently suffer from the condition. Among those over 50 years, one-third of women and one-fifth of men experience osteoporotic fractures (OPF), with a new fracture occurring every 3 s, and 50% of osteoporotic fracture patients will experience a recurrence (1, 5–7). Osteoporosis is caused by multiple factors, including estrogen deficiency, aging, genetics, calcium loss, long-term glucocorticoid use, and smoking or alcohol abuse (8, 9). Currently, diagnosis primarily relies on imaging modalities such as X-ray, computed tomography (CT), and magnetic resonance imaging (MRI) (10, 11). However, diagnostic efficacy is limited by image noise and individual variability. Treatments include bone resorption inhibitors and bone formation promoters, which have drawbacks such as side effects, limited long-term effectiveness, and the ability only to slow disease progression, preventing fractures but not reversing the condition (12, 13). The utilization of blood biomarkers, gene expression analysis, and predictive modeling can significantly enhance the diagnosis of osteoporosis, the monitoring of treatment efficacy, and the investigation of novel mechanisms and targeted therapies (14, 15). Given that early-stage osteoporosis is often asymptomatic, it is essential to integrate imaging, biochemical markers, genetic data, and clinical parameters into a comprehensive, multidimensional, and multimodal assessment framework.

Artificial intelligence (AI) was formally proposed by John McCarthy in 1956 (16). Recent advancements in technologies such as cloud computing and big data have enabled significant progress in osteoporosis research (17). The selection of AI algorithms for osteoporosis research needs to be based on data scale, feature types, interpretability requirements, and computational resources. Depending on the application scenarios, osteoporosis research primarily involves the fields of data science and image processing within AI (17–19). The identification of biomarkers through feature engineering and the application of algorithms such as support vector machines (SVM), gradient boosting machines (GBM), random forests (RF), and least absolute shrinkage and selection operator (LASSO) represents an effective approach for both diagnosing osteoporosis and advancing drug development for the condition. Further, deep neural network (DNN) technology, based on deep learning algorithms, enables precise classification and detection of medical images, as well as the prediction of skeletal diseases through hierarchical feature extraction. Notably, AI models integrating multimodal data can assess the progression of knee joint diseases, provide comprehensive technical support for the prevention of osteoporotic fractures and personalized diagnosis and treatment of bone metabolic disorders, and contribute to the advancement of osteoporosis drug development (20–22).

This paper synthesizes key advancements in AI applied to bone image recognition, pathological mechanism analysis, clinical decision support, and drug development. It presents a comprehensive review of AI research progress in the prediction, diagnosis, treatment, and novel drug development for osteoporosis. The analysis focuses on efficient target identification and efficacy assessment in drug discovery, disease risk modeling for preventive strategies, breakthroughs in medical imaging recognition for diagnostic applications, and the optimization of individualized intervention plans. Moreover, this paper highlights the potential of artificial intelligence to enable precise management of bone metabolic disorders, support the development of innovative prevention strategies, and deliver intelligent enhancements to existing healthcare systems.

## 2 Application of AI in the foundational research of osteoporosis

AI has advanced osteoporosis foundational research through multi-omics data integration and enhanced machine learning algorithms, achieving efficient target screening and drug development (23–25). Training models with multidimensional datasets has enabled the identification of crucial diagnostic markers, including serum proteins, metabolic markers, and cell death regulators, revealing core mechanisms within the metabolism-immune-cell death network. AI utilizes molecular biomarkers for data analytics, further accelerating drug development via target identification and clinical trial compound prediction.

### 2.1 Novel diagnostic molecular markers of osteoporosis

**Figure 1:**
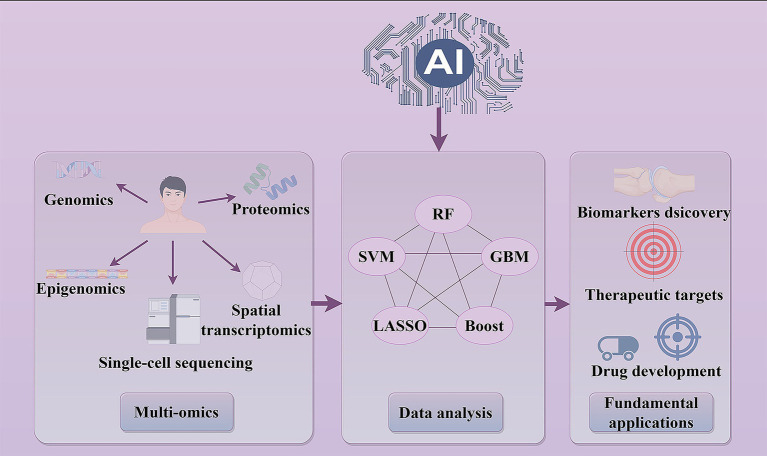
AI advances understanding of osteoporosis biomarkers and drug discovery. AI has significantly advanced foundational research in osteoporosis, elucidating disease mechanisms and enhancing drug development processes. By integrating multi-omics data with machine learning algorithms such as random forest (RF), support vector machine (SVM), gradient boosting machine (GBM), and least absolute shrinkage and selection operator (LASSO), AI facilitates the identification of crucial molecular biomarkers and target genes implicated in osteoporosis. By optimizing the exploration of pharmacological mechanisms by deep learning, AI contributes to the development of novel therapeutics and refines drug screening processes, thereby driving innovation in therapeutic strategies.

At present, AI can efficiently identify molecular markers by integrating multi-omics data, thereby significantly improving the efficiency of target identification (26). The identification of new molecular markers for osteoporosis relies on advanced computational methodologies. AI algorithms can be classified into supervised learning, unsupervised learning, semi-supervised learning, and reinforcement learning (27, 28). According to the multi-dimensional data characteristics, the identification of new diagnostic molecular markers mostly adopts supervised learning algorithms. This section focuses on the multi-dimensional research innovations and technological advancements in molecular marker screening with AI (Figure 1).

#### 2.1.1 AI identifies primary osteoporosis biomarkers

AI significantly enhances the precision of molecular biomarker discovery by integrating multidimensional data from genomics, proteomics, and metabolomics, addressing the dimensional limitations inherent in traditional analyses (Table 1). The systematic analysis of the target functional network indicates that osteoporosis involves multiple pathways related to immunity, cell death, and metabolism.

**Table 1:** AI enhances the comprehension of osteoporosis molecular biomarkers.

Data source	Algorithm	Biomarkers	Reference
Peripheral blood mononuclear cells	Support vector machine, similarity network fusion and consensus clustering	GPR31, GATM, DDB2, ARMCX1, COQ9, etc.	(29)
Peripheral blood mononuclear cells, peripheral blood B cells	Least absolute shrinkage and selection operator, support vector machine-recursive feature elimination	CCR5, IAPP, IFNA4, IGHV3-73, PTGER1	(30)
Peripheral blood mononuclear cells	Least absolute shrinkage and selection operator, random forest, support vector machine-recursive feature elimination	JUN, HMOX1, CYSLTR2, TNFSF8, IL1R2, SSTR5	(31)
Serum proteome	Light gradient boosting machine	PHLD, SAMP, A2MG, APOA1, SHBG, etc.	(32)
Plasma proteome	Light gradient boosting machine	FSHB, SOST, ADIPOQ, CKB, etc.	(33)
Peripheral blood mononuclear cells	Least absolute shrinkage and selection operator, support vector machine-recursive feature elimination	DAP3, BIK, ACAA2	(34)
Peripheral blood mononuclear cells	Support vector machine-recursive feature elimination, random forest	FOXO3, DDIT3	(35)
Peripheral blood mononuclear cells	Support vector machine, random forest	HMOX1, HAMP, LPIN1, MAP3K5, FLT3	(36)
Peripheral blood mononuclear cells	Boruta, least absolute shrinkage and selection operator, random forest	PDPK1, MAP1LC3B, ZFP36, DRAM1, MPO	(37)
Randall’s plaque, peripheral blood Mononuclear cells	Boruta, least absolute shrinkage and selection operator	WNT1, AKT1, TNF	(40)
peripheral blood mononuclear cells, bone marrow mesenchymal stem cells	Extreme gradient boosting	IGKC, TMEM187, RPS11, IGLL3P, GOLGA8N	(41)
Peripheral blood mononuclear cells, peripheral blood	Least absolute shrinkage and selection operator	HDAC6, IL-8, PPIF	(42)
Ligamentum flava	Least absolute shrinkage and selection operator, support vector machine-recursive feature elimination	SERPINE1, SOCS3, AKT1, CCL2, IFNB1	(43)
Peripheral blood mononuclear cells, peripheral blood B cells, bone marrow mesenchymal stem cells	Support vector machine-recursive feature elimination, least absolute shrinkage and selection operator, random forest, gradient boosting machine, extreme gradient boosting	ROCK1, KCNJ2, HIPK1	(44)
Peripheral blood mononuclear cells, liver	Support vector machine-recursive feature elimination, gradient boosting machine-recursive feature elimination, random forest-recursive feature elimination, least absolute shrinkage and selection operator	USP10, ERAL1, ECM1	(45)
Peripheral blood mononuclear cells, human mesenchymal stem cells	Stepglm + Gradient boosting machine	CKM, SOAT2, SGK1, ERAP2, MMP12	(46)
Peripheral blood mononuclear cells	Random forest, generalized linear, support vector machine, extreme gradient boosting	FOXO3, LUM	(47)
Human mesenchymal stem cells	Least absolute shrinkage and selection operator, support vector machine-recursive feature elimination, random forest	PANK2	(48)

The discovery of immune biomarkers for osteoporosis utilized supervised learning algorithms, including SVM, GBM, and linear regression methods. The SVM algorithm classifies the gene transcriptional data and identifies characteristic genes by finding the optimal separation plane. By integrating SVM algorithms and the similarity network fusion (SNF) method, ten characteristic genes were identified, such as GPR31, GATM, DDB2, ARMCX1, COQ9, and CD9, highlighting the significant role of metabolic heterogeneity in osteoporosis (29). Subsequent studies further utilized the SVM-REF algorithm combined with the RFE algorithm to eliminate unimportant features and combined LASSO algorithm to identify CCR5, IAPP, IFNA4, IGHV3-73, and PTGER1 of osteoporosis (30). Additionally, immune infiltration analysis confirmed that CCR5-mediated inflammation and bone imbalance are key drivers of osteoporosis progression. Moreover, the algorithm fusion of RF, SVM-RFE, and LASSO regression has achieved significant progress in the immune microenvironment (31). Immune-related differentially expressed genes were identified in peripheral blood mononuclear cells from PMOP patients, including JUN, HMOX1, CYSLTR2, TNFSF8, IL1R2, and SSTR5. Single-cell sequencing revealed that JUN and HMOX1 are differentially expressed in M1/M2 macrophages, highlighting the role of M2 polarization and its interactions with CD8 + T cells, Tregs, and fibroblasts in maintaining bone homeostasis.

The GBM algorithm aggregates heterogeneous data modalities into unified representations through iterative residual minimization guided by gradient descent optimization. Integrated analysis employing Light Gradient Boosting Machine (Light GBM), proteomics, and MR models identified 22 BMD-associated proteins, including A2MG, APOA1, PHLD, AOPL1, SHBG, and SAMP, with significant correlations at lumbar spine and femoral neck (32). MR analysis further substantiated the genetic causal effects of BCHE and APOL1 on bone mineral metrics, particularly revealing APOL1’s positive impact at the femoral neck, elucidating mechanistic links between serum proteome dynamics and bone mass. Similarly, the integration of Light GBM, MR, Cox proportional hazards regression, and plasma proteomics analysis enabled the discovery of 134 osteoporosis-associated plasma protein biomarkers through analysis of 42,325 prospectively enrolled UK Biobank (UKB) (33). ADIPOQ, CKB, FSHB and SOST were identified as high-potential predictors, introducing novel biomarkers for trimester screening of osteoporosis.

The discovery of osteoporosis cell death biomarkers predominantly utilized SVM and RF algorithms. As reported, Machine learning analysis with the SVM-RFE/LASSO model identified apoptosis-related biomarkers, including DAP3, BIK, and ACAA2 (34). Mitochondrial oxidative phosphorylation pathways, regulated by transcription factors such as SETDB1 and ZNF281, play a crucial role in modulating both programmed cell death and the remodeling of the immune microenvironment. The combined SVM-RFE and RF algorithms model established the connection between iron homeostasis regulation and bone matrix synthesis damage. The algorithm model identified the FOXO3/DDIT3 regulatory axis and revealed that MAPK signaling and neutrophil activation amplify bone resorption, offering new insights into ROS accumulation caused by estrogen deficiency in postmenopausal osteoporosis (PMOP) (35). In addition, SVM and RF models have been applied to identify key ferroptosis genes, including HMOX1, HAMP, LPIN1, MAP3K5, and FLT3 in PMOP (36). Moreover, the proposed model demonstrated robust predictive performance based on COL1A1 expression, linking iron homeostasis to compromised bone matrix synthesis.

To handle high-dimensional data, the researchers also combined RF, Boruta, with the LASSO algorithm, and studied the interaction network between autophagy and cell apoptosis (37). Cross-validation of datasets (GSE56814/GSE56815) identified key autophagy regulatory factors, including PDPK1, MAP1LC3B, and ZFP36. This investigation has elucidated transcription factor-mediated immune response and cell death signaling pathways, signifying a paradigm shift from single-pathway examinations to multi-modal regulatory network analyses in programmed cell death (PCD) research. Investigations integrating differential gene screening, machine learning, and molecular network construction have established a framework linking biomarker discovery to pathological mechanisms. These findings highlight the cascading roles of programmed cell death, oxidative stress, ferroptosis, and autophagy in bone remodeling and improve marker screening accuracy through optimized algorithms.

The integration of multi-omics with machine learning has overcome traditional limitations in apoptosis pathways research, revealing novel mechanistic insights into cell death regulation. The dynamic balance between programmed cell death and oxidative stress networks was clarified in bone homeostasis. Collectively, AI substantially enhances the efficiency and reliability of biomarkers discovery through feature screening processes and subtype stratification strategies.

#### 2.1.2 AI identifies secondary osteoporosis biomarkers

Recent cross-system studies of osteoporosis pathogenesis have made significant breakthroughs through the integration of AI approaches and multi-omics (38, 39). AI-driven systems biology overcomes limitations of single-omics studies and shifts from isolated pathways to integrated networks involving metabolism, immunity, and cell death, offering a new framework for studying bone remodeling. These studies have uncovered molecular networks linking osteoporosis with urinary, digestive, infectious, and immune system disorders. LASSO and SVM-RFE algorithms are currently central to biomarker identification in secondary osteoporosis.

The LASSO algorithm is employed to analyze osteoporosis data by eliminating irrelevant genetic features, thereby reducing noise and ensuring data independence. Its robustness in handling high-dimensional genomic datasets with inherent variability makes it particularly suitable for large-scale experimental studies. In urology, research utilizing the NHANES cohort has demonstrated a significant correlation between osteoporosis and osteopenia and the risk of kidney stones (40). Combining LASSO/Boruta algorithms and multi-omics analysis, pivotal regulatory genes, including WNT1, AKT1, and TNF, were identified, with the mTOR signaling pathway emerging as a shared mechanism underlying both conditions. Molecular docking studies further validated that drugs targeting mTOR exhibit dual regulatory effects, indicating potential novel therapeutic strategies for disorders related to bone metabolism and calcium deposition. Wang’s team (41) identified pivotal glutamine metabolism genes (IGKC, TMEM187, RPS11) and confirmed metabolic reprogramming mechanisms by tumor-associated pathway integration, substantiating the glutamine dependency hypothesis. Through the application of weighted gene co-expression network analysis (WGCNA) and iterative LASSO screening, they identified IGKC, TMEM187 five other core genes implicated in bone metabolism imbalance. The discovery extends the theoretical link between tumors and bone diseases and establishes a comprehensive framework from metabolic detection to targeted intervention. The discovery extends the theoretical link between tumors and bone diseases and establishes a comprehensive framework from metabolic detection to targeted intervention.

The use of LASSO for data selection and the establishment of models through the SVE-REF algorithm has become a current research hotspot. Xu’s team combined LASSO and SVE-REF, and through the application of bioinformatics, expanded the research on the underlying mechanisms of infectious diseases and osteoporosis to immune-mediated diseases (42). They were the first to reveal the connection between inflammatory bowel disease (IBD), osteoporosis and the HDAC6/IL-8/PPIF gene cluster, demonstrating that there is a cross-disease association between IBD and osteoporosis. Concurrently, advanced computational integration of protein–protein interaction networks and immune cell profiling techniques has elucidated patho-mechanistic links between ligamentum flavum ossification (OLF) and senile osteoporosis (SOP) through algorithmic unification (43). The identification of the IFNB1 regulatory axis extends SOP research into the domain of ectopic ossification, uncovering an inflammatory-immune-mineralization cascade central to degenerative osteoarticular disorders. Systems biology approaches have driven transformative advances in osteoporosis. On this basis, Lai’s team investigated PMOP through integrated application of WGCNA and machine learning algorithms (SVM-RFE, LASSO, RF, GBM, XG-Boost), establishing a methodological framework that enabled precise identification of ROCK1, KCNJ2, and HIPK1 as key targets from 1,278 candidate genes (44). ROCK1 exhibits dual pathological roles, demonstrating stability as a cross-dataset diagnostic marker while showing low-level expression correlation with activated innate immune pathways. Pan-cancer analyses reveal ROCK1’s implication in tumor progression, suggesting potential epigenetic crosstalk between bone metabolism and the tumor microenvironment, while methodologically aligning with prior glutamine metabolic gene through shared LASSO and SVM-RFE algorithm. While these studies all adopted a modeling approach combining LASSO and SVM-RFE, they focused on distinct mechanisms: one on the regulation of the immune microenvironment and the other on metabolic reprogramming. Recent studies have integrated the GBM-RFE algorithm to uncover the core pathological mechanisms underlying infectious diseases and osteoporosis. The tri-network diagnostic model for HBV infection, liver fibrosis, and bone metabolism disorders was enhanced through SVM-RFE, GBM-RFE, RF-RFE, and LASSO algorithms, identifying 16 gene biomarkers (USP10, ERAL1, ECM1) that achieved 79% diagnostic accuracy via rigorous gene screening, marking significant progress in hepatogenic bone disease research (45). By integrating omics data with algorithmic models, researchers are constructing a comprehensive knowledge network that links molecular interactions to clinical applications, thereby advancing bone metabolism disorder research beyond traditional paradigms.

#### 2.1.3 AI identifies osteoporosis biomarkers induced by environmental pollutants

The integration of AI-powered multi-omics is revolutionizing the investigation of the effects of environmental pollutants on osteoporosis. Osteoporosis biomarker identification linked to environmental pollutants primarily relies on decision tree algorithms such as Boost and RF.

XG-Boost optimizes the traditional Boost algorithm, making it faster and using less memory when dealing with large datasets. The XG-Boost algorithm, when combined with the GBM algorithm, prevents overfitting of data and maintain better performance in cases with excessive data noise. Network toxicology, integrated with the Stepglm + GBM model, identified critical genes CKM and MMP12 associated with phthalate esters (BBP/DBP/DEHP) and osteoporosis (46). Molecular docking analyses demonstrated that these pollutants interact with TRP and GLU amino acids, disrupting the IL-17/TNF signaling pathway and influencing osteoclast differentiation. Establishing the inaugural molecular pathway linking chemical-gene interactions to imbalances in bone metabolism, XG-Boost, which integrates RF and SVM algorithms, intelligently combines the results of all models, reducing the risk of overfitting and significantly improving the model’s AUC (47). Building on this foundation, Huang’s team found that environmental endocrine disruptors (EDCs) affect TNF signaling by forming hydrogen bonds with FOXO3 and LUM. Among 13 EDCs, dexamethasone, perfluorooctanoic acid, and soy isoflavones bind to FOXO3, while genistein and prednisolone interact with LUM, disrupting bone-related gene networks and providing cross-scale insights of how EDCs contribute to osteoporosis pathogenesis.

The RF algorithm integrates SVM-RFE and LASSO for feature selection, enabling systematic identification of the optimal feature subset (48). This approach was applied to identify PANK2 as a key mediator in PFAS-induced osteogenesis. Also, simulations showed stable hydrogen bonds between PFOS and PANK2, and experiments confirmed its role in regulating chemokine pathways via miR-26a-5p, leading to imbalances in BMSCs’ osteogenic and lipogenic differentiation. This integrative methodology, encompassing molecular docking, machine learning, and experimental validation, demonstrates that while various pollutants engage distinct molecular mechanisms (CKM/MMP12, FOXO3/LUM), they collectively perturb osteogenesis, osteoclast homeostasis, and the equilibrium between osteogenesis and lipogenesis. These insights provide potential biomarkers for the early detection of osteoporosis and establish a foundation for developing targeted therapies and strategies for environmental risk mitigation. In terms of identifying osteoporosis molecular markers, the adoption of various algorithms to improve the model has become the current trend. The strategic integration of LASSO for data preprocessing, Boost algorithms for memory optimization, and ensemble methods (RF/SVM/GBM) for multi-perspective feature selection significantly enhances feature stability, thereby improving model performance and interpretability. These methodological advancements establish an interdisciplinary framework synergizing AI algorithms, multi-omics integration, and molecular biomarkers, catalyzing innovative approaches for osteoporosis biomarker discovery.

### 2.2 Novel drug discovery for osteoporosis

Artificial intelligence has demonstrated significant potential in advancing osteoporosis therapeutics (49, 50). Drug discovery-driven innovative modeling approach, synergized with multi-source data integration, has substantially improved screening efficiency and reduced development timelines (51, 52). Furthermore, AI facilitates the optimization of clinical trial design and accelerates the overall drug development pipeline (Table 2).

**Table 2:** AI enhances osteoporosis drug discovery.

Data source	Algorithm	Agents	Targets	Reference
Drug signatures database	Random forest, support vector machine-recursive feature elimination	Geniposide	HMOX1	(55)
Comparative toxicogenomic database	Least absolute shrinkage and selection operator, support vector machine-recursive feature elimination, Boruta	Vinclozolin, Bisphenol A, Benzo(a) pyrene, Valproic Acid	MAP3K3, SFSWAP, NELFB	(56)
Drug gene interaction database	Least absolute shrinkage and selection operator, support vector machine-recursive feature elimination, random forest	Bumetanide, Elacestrant	SLC12A2, ESR1	(57)
Drug Gene Interaction Database	Least absolute shrinkage and selection operator, support vector machine, random forest	Hesperadin, Melagatran	BRSK2, VPS35	(58)
Target Mol Library	Deep learning – based efficacy prediction system	Dihydroartemisinin	H3K9	(59)
Drug Bank, PubChem	Deep transformer, OPGraph, graph neural network	Puerarin, Aucubin	N/A	(60)
Traditional chinese medicine integrated ontology database	Deep screening	YYH flavonoids	HSD17B2	(61)
PubChem	Chemical property prediction	Quercetin, γ-Linolenic acid, and Benzyl isothiocyanate	CTSK	(62)

Drug discovery processes established machine learning algorithms that retain their efficacy in data analysis, while AI has achieved comprehensive integration throughout pharmaceutical development, spanning target identification, absorption distribution metabolism excretion (ADMET) prediction, and clinical trial execution (53). The RF/SVM-SFE algorithm-powered model emerges as a robust methodology for both core gene discovery and small molecule compound recognition. The integration of LASSO, SVM, and RF algorithms has identified BRSK2 and VPS35 as core genes associated with programmed cell death (54). By combining network toxicology with RF/SVM-SFE algorithms, MR, and single-cell sequencing, it was demonstrated that HMOX1-positive macrophages promote M2 polarization through the ANXA1/MIF signaling axis, while molecular docking analyses validated the binding potential of geniposide (55). Further, the algorithm integrating SVM-RFE, LASSO, and WGCNA establishes a multi-level model for analyzing transcriptomics and proteomics data. It has validated mitophagy core genes, NELFB, SFSWAP, and MAP3K3, and predicted four potential therapeutic agents: Vinclozolin, Bisphenol A, Benzo(a)pyrene, and Valproic acid (56). The model also investigated the link between endoplasmic reticulum stress (ERS) and mitochondrial dysfunction (57). Combining RF with these methods identified five key genes, AAAS, ESR1, SLC12A2, TAF15, and VAMP2, enriched in lipid metabolism, calcium transport, and ossification pathways. Bumetanide and elacestrant were further screened as candidate therapeutics via qRT-PCR and molecular docking.

Recently, deep learning models have also been applied to drug development. These models directly learn multi-level features from raw data, optimizing the exploration methods of pharmacological mechanisms. Network pharmacology was combined with DeepPurpose to show that salvianine affects bone metabolism via the CASP3/CTNNB1/ERBB2 pathways (58). Drug-target networks were predicted using DGIdb, revealing that BRSK2 binds to hesperadin and VPS35 interacts with melagatran, offering new insights into targeted therapies. The model was further refined through the development of a deep learning efficacy prediction system (DLEPS), which found that dihydroartemisinin enhances the stemness of BMSCs via GCN5-mediated H3K9 acetylation and led to the creation of a bone-targeted nano-delivery system that improves therapeutic outcomes (59).

The graph neural network model based on deep learning further predicts the connections among diseases, targets, and drugs by constructing a deeper graph network. The development of the Deep Transformer model employing graph neural networks has demonstrated remarkable efficacy (AUC = 0.9916) in predicting the bioactivities of puerarin and aucubin (60). By integrating chemical informatics with deep learning methodologies, the researchers have established a comprehensive pharmacological network for anti-osteoporosis herbal medicines, successfully identifying 89 active ingredients and 30 target genes (61). The study substantiates the roles of phytosterols, flavonoids, and alkaloids as key active components and confirms the interaction between YYH flavonoids and HSD17B2. Furthermore, the application of Chemprop’s graph neural network (AUC = 0.93) and molecular docking techniques facilitated the identification of potent CTSK inhibitors, including quercetin, *γ*-linolenic acid, and benzyl isothiocyanate (62). These compounds exert their inhibitory effects on CTSK by stabilizing the catalytic triad (Cys25/His162/Asn162) through hydrogen bonding. The development of new drugs for osteoporosis can utilize both established machine learning methods and deep learning methods. Established machine learning methods ensure the accuracy of gene and target characteristics, enabling drug development even with limited data. In deep learning algorithms, graph neural network algorithms integrate multi-omics data and can extract gene and target features from vast databases, demonstrating significant potential in advancing drug development and translational medicine. AI has achieved significant breakthroughs in molecular biomarkers, molecular mechanisms, and targeted drug development. The iterative research framework encompassing “target discovery,” “molecular mechanism,” and “clinical application” is accelerating paradigm innovation in translational medicine.

## 3 AI-driven strategies for osteoporosis management: clinical innovations and challenges

**Figure 2:**
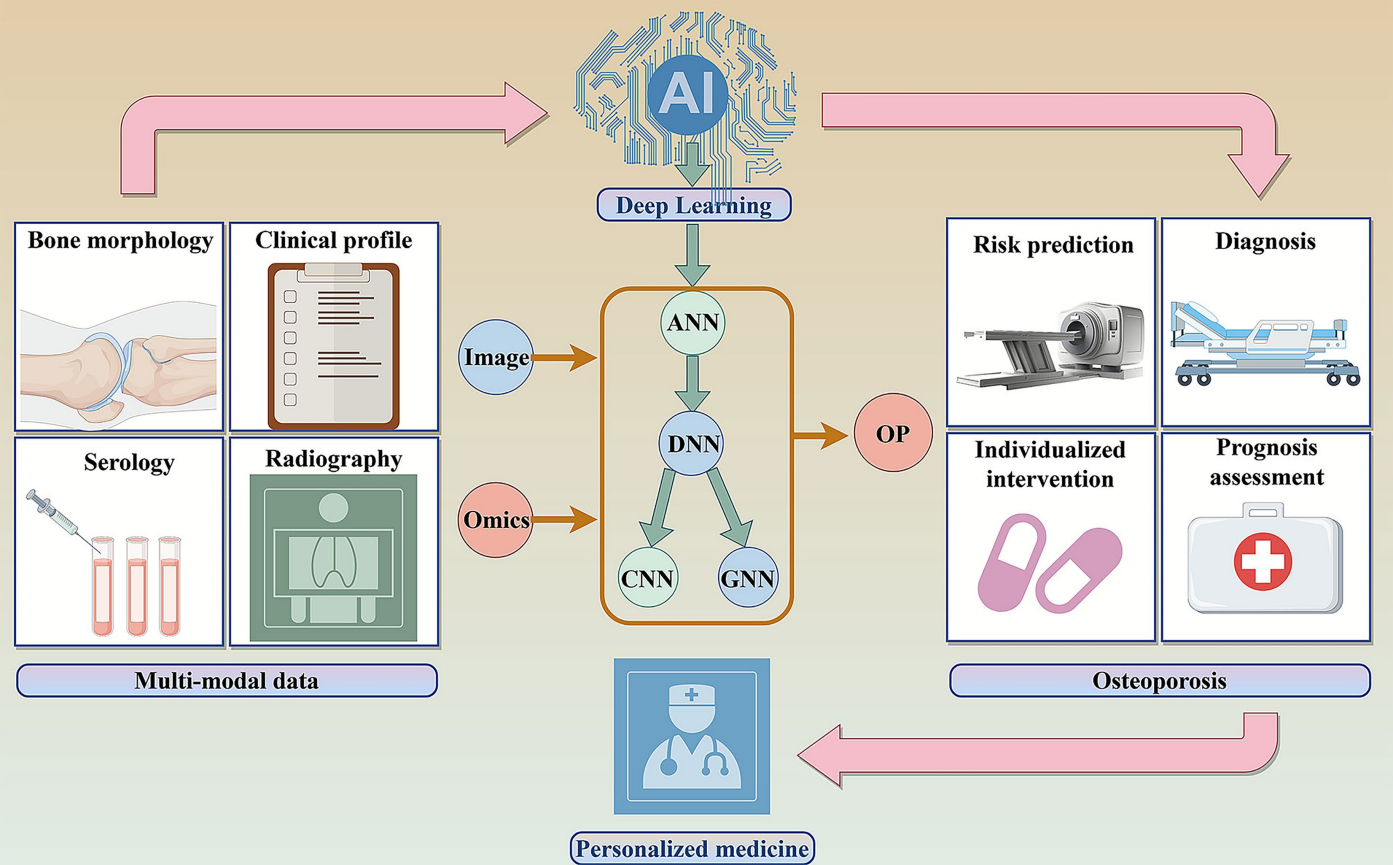
AI improves osteoporosis diagnosis and treatment. AI integrates multimodal imaging, bone morphology, clinical profiles, and serology with risk assessment frameworks to enhance osteoporosis risk stratification, facilitate precise diagnosis, and enable proactive warning systems. Through deep learning architectures, including Artificial Neural Networks (ANNs), Deep Neural Networks (DNNs), Convolutional Neural Networks (CNNs), and Graph Neural Networks (GNNs). AI synthesizes personalized therapeutic regimens. By incorporating surgical prognosis prediction and drug efficacy assessment, AI establishes a comprehensive intelligent management system encompassing osteoporosis screening, intervention, and long-term monitoring.

Osteoporosis, a metabolic bone disease associated with high disability and mortality, is benefiting from major advances in early risk screening and diagnosis through AI (63, 64). AI-driven models have surpassed the sensitivity of traditional imaging and are transforming the diagnosis of osteoporosis and fracture risk through intelligent multimodal imaging analysis. They fuse cross-modal data (CT/MRI/X-ray) with deep learning and optimize interpretable features (65–67). By integrating biomechanical simulations and reinforcement learning, these systems accurately predict bone strength and assess fracture risk, enabling an intelligent diagnostic process that connects imaging, biomechanics, and clinical decisions (Figure 2).

### 3.1 Application of AI in osteoporosis diagnosis

AI is reshaping osteoporosis screening systems by advancing image processing in osteoporosis diagnosis through deep feature extraction based on artificial neural network methods (Table 3). Meanwhile, researchers overcome DXA scan limitations by developing multimodal imaging-based intelligent diagnostic frameworks (68, 69).

**Table 3:** Application of AI in the risk prediction and diagnosis of osteoporosis.

Data source	Data size		Algorithm	Measure (internal/external)				Reference
	Internal test	External test		Accuracy (%)	AUC (95% CI)	Specificity (%)	Sensitivity (%)	
Hip radiographs imaging	800	101	Deep neural network	81.2/71.8	0.867/0.700	68.9/38.7	91.1/83.7	(71)
Spine X-ray imaging	702	176	Frequency channel-wise transformer network	78.29/55.26	0.8718/N/A	88.92/76.99	69.72/53.06	(72)
Chest radiographs and dual energy X-ray absorptiometry	13,026	1,089	OsPor-screen model	82.40/77.69	0.91/0.88	81.45/74. 19	84.31/86.16	(73)
Pelvic X-ray imaging	3,972	1,055	Convolutional neural network	N/A/84	N/A/0.893	82/87	80/76	(74)
Dual-energy X-ray absorptiometry and hand radiographs imaging	533	136	Deep learning	82.00/94.37	0.7400/0.9525	61.00/97.05	87.03/93.45	(76)
Hip, spine X-ray imaging	3,306, 1,076	876, 290	Deep learning	91.7, 86.2/91.4, 87.6	0.97, 0.92/0.96, 0.92	94.9, 88.3/94.7, 90.5	80.2, 83.5/78.9, 80.6	(78)
Knee X-ray imaging	489	122	Superfluity deep learning	83.74/74.51	0.8116/0.6883	86/79.33	76.33/58.33	(79)
Biplanar X-ray radiography	725	181	Hybrid deep learning framework	93/96	0.97/0.97	96/97	84/88	(82)
Spine X-ray imaging, lumbar and femoral dual energy X-ray absorptiometry	100	74	Artificial neural network	79.77/79.34	0.781/0.846	73.44/82.93	86.11/75.76	(90)
Hand radiographs imaging	7,764	2,587	HarDNet-based deep learning	N/A/80	N/A/0.883	N/A/83	N/A/73	(93)

The artificial neural networks (ANN) method eliminates the need for manual feature design and has broad applications in osteoporosis diagnosis. For example, existing research combines ANN with ultrasound frequency-dependent attenuation to estimate microstructural parameters of cortical bone, such as pore diameter (*φ*), pore density (*ρ*), and porosity (*ν*) (70). The DNN algorithm, built upon the foundation of ANN, enables the extraction of complex and abstract features, making it particularly well-suited for analyzing intricate bone imaging data and more closely aligned with real-world clinical applications. Leveraging the DNN algorithm, a model incorporating hip joint images and Grad-CAM visualization achieved an accuracy of 71.8%, sensitivity of 83.7%, specificity of 38.7%, and an AUC of 0.700 (71). To address the challenges posed by high noise and low contrast in X-ray images, an innovative Frequency Channel-Wise Transformer Network (FCoTNet) was developed, integrating multi-scale feature extraction and convolutional fusion across diverse receptive fields (72). The model achieved an AUC of 0.8718, with accuracy, sensitivity, and specificity reaching 78.29, 69.72, and 88.92%, respectively. Furthermore, it enhanced clinicians’ diagnostic performance by 18.5% in accuracy, 18.93% in sensitivity, and 9.83% in specificity. Multimodal data fusion has emerged as a promising strategy to improve diagnostic efficiency.

Convolutional neural networks (CNNs) are a specialized type of DNN designed for grid-structured data like medical images. They are better suited for bone image analysis than conventional DNNs and are less prone to overfitting. Leveraging these advantages, researchers have developed intelligent diagnostic systems using multimodal imaging that surpassed the limitations of DXA scans. Researchers assessed bone density and demonstrated robust cross-racial performance, with an AUC of 0.88, accuracy of 77.69%, specificity of 74.19%, and sensitivity of 86.16%, indicating the potential for a more universally applicable screening approach (73). Breakthroughs in advanced 3D image analysis and micro-parameter measurement technologies have enabled the development of a multi-tier CNN architecture utilizing lumbar X-ray images, significantly enhancing opportunistic CT screening capabilities. A CNN-based DeepDXA model demonstrates high accuracy in estimating bone mineral density from pelvic X-ray images, exhibiting strong predictive performance with an accuracy of 84%, sensitivity of 76%, specificity of 87%, and an AUC of 0.893 (74). Advances in AI have driven the development of innovative osteoporosis diagnostic methods, with model interpretability emerging as a critical factor for clinical adoption (75). As reported (10), researchers improved a deep U-Net-based diagnostic algorithm that integrates softmax prediction with a cross-entropy loss function to construct an energy function, enabling more accurate bone mineral density measurement with an automatic recognition rate exceeding 81%, demonstrating strong clinical potential. A deep learning model combining hand X-ray and DXA images achieved 94.37% accuracy, 93.45% sensitivity, 97.05% specificity, and an AUC of 0.9525, showing potential for early screening (76).

Model interpretability has become a key factor in clinical adoption (77, 78). By integrating Grad-CAM technology, a CNN-based HarDNet deep learning model was introduced for non-invasive bone mineral density prediction using hand X-ray images, enhanced interpretability through heat map visualization, achieving a sensitivity of 78.9, 80.6, specificity of 94.7, 90.5, accuracy of 91.4, 87.6, and AUC of 0.96, 0.92, showing promise for early screening, showing promise for early screening. Superfluity deep learning model analyzing knee X-ray images achieved an AUC of 0.6833, an accuracy of 74.51%, and a specificity of 79.33%, demonstrating potential for multi-site skeletal evaluation (79). Further, advances in AI have led to the development of innovative methods for osteoporosis diagnosis (80). An improved DenseNet121 model was applied to perform 3D segmentation and predict bone mineral density of T12, L1, and L2 vertebrae using low-dose chest CT images. Bland–Altman analysis confirmed the system’s high performance, with sensitivity over 86%, specificity over 98%, and an accuracy rate of 86.6%. The system is compatible with images from different scanners and unaffected by age or gender, demonstrating strong cross-device stability and broad applicability. A model using CT attenuation values showed that each 10 HU increase was associated with a 32–44% lower risk of osteoporosis (AUC = 0.831), with particular relevance for menopausal women (66). New algorithm architectures have improved the fusion of clinical information in data integration. Another study applied graph neural networks (GNN) to model personal health and lifestyle data as topological structures, enabling dynamic risk assessment and personalized prevention strategies (81).

The hybrid deep learning framework (HDLF) combines various types of ANN algorithms and enhances the integration of clinical information in data consolidation. Research combined pelvic X-rays, vertebral data, and clinical variables to predict BMD and classify disease with high accuracy (AUC 0.97, accuracy 96%, specificity 97%, sensitivity 88%) (82). Notably, this approach was further refined in later research, where a generalized additive model (GA^2^M) identified that the interaction between spinal BMD and body weight was a key predictor of 10-year osteoporosis risk (AUC 0.83) (83). On model interpretability, researchers used SHAP/LIME/ELI5 tools to analyze a multi-layer ensemble model and found that low femoral neck and thoracolumbar T values (≤ − 2.5) were major risk factors, with SHAP values strongly negatively correlated with disease risk (84). According to research findings (71, 82), models developed using the DNN algorithm demonstrate higher sensitivity but relatively lower accuracy, whereas those utilizing the HDLF algorithm achieve the highest accuracy. The SHAP tool has high computational complexity, resulting in significant computational costs for large models. The LIME tool requires subjective selection of kernel functions and kernel width, which can significantly affect the interpretation results, as different settings may highlight different features. Compared to SHAP and LIME, ELI5 has relatively weaker interpretability capabilities for complex data such as images and text.

Accurate diagnosis of osteoporosis constitutes a critical component of its clinical management, offering substantial value in slowing disease progression and preventing fragility fractures (85). Within the domain of imaging diagnostics, intelligent diagnostic approaches are advancing osteoporosis assessment toward enhanced precision and efficiency. As more clinical evidence emerges, AI-assisted diagnostic systems are poised to play a central role in osteoporosis management.

### 3.2 Application of AI in osteoporotic fracture risk assessment

Osteoporotic fractures represent the most common complication of osteoporosis and a leading cause of disability and mortality (86). The application of AI in osteoporosis risk assessment is transforming traditional diagnostic paradigms towards greater precision. Future research should emphasize its role in fracture risk evaluation to strengthen clinical decision support (87). The Fracture Risk Assessment Tool (FRAX) system is the most widely adopted tool for global fracture risk assessment, providing a ten-year probability model based on clinical factors to enable effective risk stratification (88). With advances in deep learning, intelligent assessment leveraging medical imaging has evolved from qualitative interpretation to quantitative prediction.

By integrating hip and spine X-ray images with an ANN model, researchers achieved high diagnostic accuracy for hip osteoporosis (91.7%), lumbar osteoporosis (86.2%), 10-year osteoporotic fracture prediction (95%), and high-risk hip fractures (90%) (89). These results showed no significant difference compared to the FRAX system, confirming the reliability of AI in standardized risk assessment. The development of dynamic risk models introduces a novel temporal dimension for disease monitoring. The integration of TWIST and ANN neural networks with a femoral bone strain index (Femoral BSI) demonstrated a specificity of 82.93%, sensitivity of 75.76%, accuracy of 79.34%, and an AUC of 0.846 in identifying vertebral fractures among postmenopausal women (90). Compared to the conventional ANN algorithm, the improved DNN algorithm employing DeepHit enables non-parametric data analysis without reliance on predefined distribution assumptions. A dynamic scoring system combining DeepHit and Cox proportional hazards models successfully predicted hip fracture risk in a discovery cohort, achieving a Harrell’s C index of 0.860 and accurately forecasting a 10-year incidence of severe fractures at 68.8%, thereby significantly enhancing early warning capabilities for critical fracture events (91). A deep learning model for vertebral fracture classification based on spinal X-rays attained an AUC of 0.948, with a sensitivity of 54.5%, a specificity of 99.7%, and an accuracy of 89.8% in independent testing (92). An AUC-ROC exceeding 0.90 confirms its diagnostic performance, which is comparable to that of experienced radiologists.

The integration of radiomics and 3D modeling has expanded the applicability of CNN algorithms in bone morphological analysis. A CNN-based thoracolumbar spine CT analysis system achieved 83.9% accuracy in identifying compression fractures by extracting discriminative imaging features (AUC = 0.883) (93). A 3D CT-based dynamic prediction model for hip fractures, built on the DenseNet architecture, maintained an AUC of 0.73–0.74 over 1 year, outperforming conventional clinical prediction models (94). For special populations, multi-omics data fusion is enabling more precise preventive strategies. A gradient boosting model integrating genetic risk scores achieved 88% prediction accuracy in elderly men (AUC = 0.71), surpassing the FRAX method and demonstrating the value of multi-omics integration in personalized risk assessment (95). Opportunistic screening using routine imaging data provides essential technical support for establishing a tiered diagnosis and treatment framework. Further optimization led to the development of a GoogLeNet-based model for fracture severity grading, designed specially to identify moderate to severe fractures (96). This model achieved an AUC-ROC of 0.99 and an area under the precision-recall curve (AUPRC) of 0.82, balancing a high positive predictive value (91.2%) with moderate sensitivity (59.8%). Through optimized probability thresholds, it minimizes unnecessary interventions. This hierarchical early warning mechanism establishes the algorithmic foundation for automated screening, marking a significant advancement toward the clinical implementation of AI-assisted diagnostic systems.

### 3.3 Application of AI in osteoporosis treatment and prognosis assessment

Improving the precision of pharmacological interventions and optimizing prognostic evaluation remain key priorities in clinical research (97). Notably, AI exhibits significant potential in predicting disease progression, developing personalized treatment strategies, managing postoperative care, and assessing drug efficacy via its advanced data processing and pattern recognition capabilities (98, 99).

AI also supports the development of personalized treatment strategies and offers broad applications in osteoporosis care (100). An RF model utilizing 8,981 clinical variables achieved an AUC of 0.70 and an accuracy of 69% in predicting responses to 11 distinct treatments (101). The model demonstrated a 9.54% improvement over observed clinical outcomes, thereby reinforcing the potential for more individualized therapeutic approaches. Further, a clinical decision support system that integrated DXA results with clinical data through hierarchical feature extraction and novel rule-based evaluation methods achieved decision accuracy of 90%, providing an actionable strategy for prevention and treatment planning (102). Different machine learning algorithms exhibit varied efficacy in distinct applications. Comparative analyses have revealed that among ANN, RF, SVM, and LR models, the RF model achieved the highest accuracy at 75.0%, while the LR model yielded the highest AUC at 0.731. These findings indicate that algorithm selection should be tailored to specific clinical objectives and evaluation metrics (103). This principle is further validated by a multimodal evaluation framework that elucidated the regulatory mechanisms of the miRNA-m6A network and accurately predicted drug sensitivity to clozapine and aspirin by identifying key genes DEFA4 and HLA-DPB1 using the SVM algorithm (104). Intelligent algorithms have demonstrated substantial efficacy in postoperative prognosis management (105). An RF model with an AUC of 0.953 demonstrated superior performance compared to traditional logistic regression (AUC = 0.831) in predicting vertebral fracture recurrence among 529 patients undergoing percutaneous kyphoplasty (PKP), highlighting the potential of machine learning to enhance surgical decision-making (106). Moreover, an SVM classifier, achieving an AUC of 0.85, accuracy of 81%, and a sensitivity of 98%, effectively identified multidimensional risk factors for PKP recurrence through multivariate logistic regression, establishing a quantitative foundation for preventive interventions (107). Deep learning is increasingly employed in the assessment of pharmacological efficacy. An ANN analysis of spinal radiographs and bone strength parameters revealed that teriparatide enhanced BSI by 13.9%, trabecular texture bone score (TBS) by 5.08%, and BMD by 8.36%, thereby demonstrating its effectiveness in mitigating fracture risk (108). AI is transforming osteoporosis diagnosis and treatment, from risk assessment and diagnosis to clinical solution optimization and efficacy evaluation by creating an intelligent decision support network. As algorithms integrate with multimodal data, intelligent systems are enhancing clinical decision accuracy and advancing personalized, dynamic management of osteoporosis.

## 4 Conclusion and perspective

AI transforms healthcare by integrating multimodal data and advanced technologies. In diagnosis, a combination of multi-omics data, including genomics, proteomics, metabolomics, and radiomics, is utilized to build dynamic visual pathological models that enable disease risk stratification and early warning systems. In clinical decision-making, intelligent monitoring systems analyze biomolecules and radiomic features to create a virtual-real integrated diagnosis and treatment ecosystem with predictive intervention. Furthermore, machine learning and network pharmacology models enhance the understanding of the molecular biomarkers and pathological mechanisms underlying osteoporosis. Deep learning-driven molecular dynamics simulations accelerate new drug discovery. The AI system has established a novel digital precision medicine paradigm by integrating biomarker monitoring, clinical imaging analysis, personalized medication plans, and drug innovation while optimizing clinical decisions to advance osteoporosis diagnosis and treatment toward enhanced precision and intelligence.

Nonetheless, while AI holds significant potential in clinical applications, it still carries certain limitations. Regarding data standardization, different data subsets are influenced by gradient descent algorithms during preprocessing, which can easily induce model drift and lead to degraded model performance. In terms of interpretability, algorithmic bias means that some training data may not represent the target population, resulting in reduced diagnostic accuracy. From an ethical standpoint, the clinical application of artificial intelligence raises significant concerns regarding patient data privacy. These challenges pose substantial barriers to the regulatory approval of AI-integrated healthcare technologies. Future research should foster collaboration between AI researchers and clinicians to integrate AI findings into clinical workflows, improving model performance and interpretability. Incorporating emerging approaches such as federated learning and multi-modal data coordination, and addressing real-world integration challenges can help resolve ethical concerns in the clinical use of AI.

## Glossary

ADMET: absorption distribution metabolism excretion
AI: artificial intelligence
ANN: artificial neural network
ANXA1: Annexin A1
AUC: area under the curve
AUPRC: area under the precision-recall curve
BMD: bone mineral density
BPX: biplanar X-ray images of the pelvis
BSI: bone strain index
CASP3: Cysteine aspartic acid protease 3
CNN: convolutional neural networks
CT: computerized tomography
CTSK: Cathepsin K
DL: deep learning
HDLF: hybrid deep learning framework
DLEPS: deep leaning-based therapeutic effect prediction system
DNN: deep neural network
DXA: dual-energy x-ray absorptiometry
EDCs: Endocrine disrupting chemicals
ERS: endoplasmic reticulum stress
ERAP2: endoplasmic reticulum aminopeptidase 2
FCoTNet: Frequency Channel-Wise Transformer Network
FOXO3: Forkhead box protein O3
FRAX: Fracture Risk Assessment Tool
GA2M: generalized additive model
GBM: gradient boosting machines
GNN: graph neural network
HMOX1: Heme oxygenase 1
IBD: inflammatory bowel disease
IOF: international osteoporosis foundation
HSD17B2: Estradiol 17-beta-dehydrogenase 2
LASSO: least absolute shrinkage and selection operator
LR: logistic regression
Light GBM: Light Gradient Boosting Machine
MAE: mean absolute error
MAPK: Mitogen activated protein kinases
MD: mitochondrial dysfunction
MIF: Macrophage migration inhibitory factor
ML: machine learning
MMP12: Matrix metalloproteinase 12
MR: Mendelian randomization
MRI: magnetic resonance imaging
OLF: ossification of ligamentum flavum
OP: osteoporosis
OPF: osteoporotic fracture
PANK2: Pantothenate kinase 2
PCD: programmed cell death
PFAS: Perfluoroalkyl sulfonate
PFOA: Perfluorooctanoic acid
PFOS: Perfluorooctane sulfonates
PKP: percutaneous kyphoplasty
PMOP: postmenopausal osteoporosis
P-SAMPNN: pre-trained self-attentive message passing neural network
RA: rheumatoid arthritis
RF: random forest
RFE: recursive feature elimination
ROC curve: receiver operator characteristic curve
SNF: similarity network fusion
SOP: senile osteoporosis
SOST: sclerostin
SVM: support vector machine
TBS: trabecular bone score
TNF: tumor necrosis factor
UKB: UK Biobank
WGCNA: weighted gene co-expression network analysis
XG-Boost: eXtreme Gradient Boosting.
